# The subjective experiences of students with invisible disabilities at a historically disadvantaged university

**DOI:** 10.4102/ajod.v11i0.932

**Published:** 2022-06-10

**Authors:** Carushca de Beer, Serena Isaacs, Cameron Lawrence, Gugulethu Cebekhulu, Jade M. Morkel, Jonathan Nell, Noluthando Mpisane, Wayne P. van Tonder, Yolanda R. Mayman, Lobisa Z. Thobejane, Athena Pedro

**Affiliations:** 1 Department of Psychology, Faculty of Community and Health Science, University of the Western Cape, Cape Town, South Africa

**Keywords:** invisible disability, higher education, awareness, support, policy, stigma, historically disadvantaged university, disability units

## Abstract

**Background:**

Despite policies that promote inclusivity of students with various challenges, students with invisible disabilities at higher learning institutions may encounter various levels of stigma and marginalisation. This primarily stems from a lack of awareness about what invisible disabilities encompass, and very importantly, how they affect those who live with them.

**Objective:**

This study explored the subjective experiences of students with invisible disabilities at a historically disadvantaged university.

**Method:**

This study used a qualitative approach to facilitate the exploration of the subjective experiences of students with invisible disabilities. Twelve students with invisible disabilities were interviewed online via Google Meet platform, using semi-structured interviews. Interviews were transcribed verbatim and analysed using a thematic analysis.

**Results:**

Three thematic domains were identified, invisibly disabled students’ subjective experiences within the context of (1) home and community, (2) university life and (3) support offered at their university.

**Conclusion:**

This study’s findings highlight the importance of awareness of invisible disabilities in higher education. Support for students with invisible disabilities, and breaking down the barriers to it, can compound better experiences in the lives of those who have invisible disabilities. Targeted awareness campaigns could contribute to more mindful learning and teaching practices and improve the overall experience of these students. This information can also be used to promote awareness of students with invisible disabilities in higher education institutions.

## Introduction

People with disabilities in South Africa make up 13% of the population, of which 5% have a severe disability (Maziriri, Madinga & Lose 2017); however, Graham et al. (2014) asserted that the estimated number of persons who have disabilities might be higher than most numerical representations. People may be faced with a more ‘immediately’ perceptible disability, such as a physical or sensory disability (Viriri & Makurumidze 2014), and some may be faced with challenges that are not immediately perceptible, such as learning difficulties, known as invisible disabilities (Venville et al. 2016). Invisible disability, as defined by the Invisible Disabilities Association (2018), encompasses psychological, neurological or physical conditions that constrain afflicted individuals’ movements, senses or activities, but which are invisible to the spectator (Cook & Clement 2019).

Invisible disabilities include an array of disabilities such as (but not exclusive to) sleeping disorders, learning disabilities, autoimmune diseases and brain injuries (Cook & Clement 2019; Dalgin & Ballini 2008; Nalavany, Crawan & Sauber 2015; Norstedt 2019). As a result of the absence of physical markers for invisible disabilities, people with invisible disabilities may have to repeatedly decide whether to disclose their disability or not (Stone 2005).Falch and Harnaes (2012) asserted that the general lack of understanding about invisible disabilities often sets up the possibility for a negative reaction or particular barriers, which may result in the individual being ‘labelled’, isolated and excluded once a disability is disclosed (Shelton & Matthews 2001).

Practices of exclusion have a deep history in South Africa. For instance, during the apartheid regime, persons with disabilities were both marginalised and excluded from society and development initiatives (Sangeeth 2016). Furthermore, people with disabilities were excluded from primary and secondary education, and these individuals were not prepared for tertiary education. However, within the democratic South Africa this has progressed towards more inclusionary practices such as policy initiatives and development. This is specifically aligned with the white paper of 2016, which emphasises a commitment to improve access, inclusion and success for students with disabilities in a higher education setting (Department of Social Development 2016; Mutanga 2017).

### The nature of disability in higher education in South Africa

In addressing the issue of educational inclusivity at the tertiary level, the Department of Higher Education of South Africa’s approach has been particularly influenced by the following legislative documents: the United Declaration of Human Rights (1948), the United Nations International Convention on the Rights of Persons with Disabilities (UNCRPD) (2006), the South African Bill of Rights (Act 108 of 1996) and the White Paper on the Rights of Persons with Disabilities (2015). The following issues have been addressed in these documents: firstly, the right of persons with disabilities to have access to tertiary education; secondly, the equality of education provided and thirdly, the issue of reasonable accommodations for those with disabilities, within the tertiary education context. These documents are in accordance with the World Health Organization’s action plan from 2014 to 2021, which is based on the human rights premise (World Health Organization 2015). The Disability Rights Charter of South Africa has guided the Department of Higher Education and Training (DHET 2018). Specifically, it states that people with disabilities have the right to mainstream education with personal assistance where necessary, assistive technology and specialised teaching (Matshedisho 2007). Institutions of higher learning, therefore, need to be proactive in their approach to be inclusive in addressing a range of barriers, such as physical structures, methods of teaching and assessment, as well as sociocultural beliefs (Morrison, Branda & Cilliers 2009).

Thus, the role and responsibility of disability units within higher education institutions is to provide students with disabilities with equal opportunities that will give them a fair chance of succeeding in their tertiary education (Mutanga 2017). There are various higher education institutions that have managed to eliminate some of the physical obstacles, including specific support services, accessible infrastructure and academic materials. Several South African universities have specialised disability units in place that work to facilitate and manage support services for students with disabilities (Pudaruth, Gunputh & Singh 2017).

However, because of the nature of invisible disabilities, these students have a unique set of challenges resulting in a unique set of experiences (Ohajunwa, McKenzie & Lorenzo 2015; Venville et al. 2016). For instance, a study conducted by Mullins and Preyde (2013) at a Canadian university reported that invisible disabilities are often seen as ‘lesser’ disabilities, as their validity becomes questionable. Consequently, Thompson-Ebanks (2014) reported that owing to lack of reasonable accommodation, there is a notable dropout rate of students with invisible disabilities such as learning disabilities within higher education institutions.

Considering that symptoms are invisible, much of what is experienced by people with invisible disabilities is often either stigmatised, misunderstood or misperceived (Cook & Clement 2019). Therefore, navigating their daily lives can prove challenging for people with invisible disabilities. According to Matthews (2009), lecturers and tutors may not be aware of differently abled students in their classrooms, adding to the challenges that these students face. Students with invisible disabilities may not receive the same level of support that their peers with visible disabilities receive (Cook et al. 2017; Kubiak 2015; Shaw 2012; Venville et al. 2016). It is therefore imperative that both flexible and visible avenues of support and accommodation are normalised so that students are aware of their availability. Students who have disabilities might experience a lack of reasonable accommodation to the extent that they drop out of higher education (Thompson-Ebanks 2014).

Although policies of inclusion appear to ease the path of students who have invisible disabilities, their subjective experience illustrates that complex challenges still exist within the accessibility to education. Although significant strides have been made to address challenges that are faced by people who have disabilities, students within education settings may continue to be disadvantaged. According to Venville et al. (2016), most of the existing support structures implemented at higher education institutions were primarily developed for students with physical and sensory impairment. This places individuals who have invisible disabilities at a disadvantage because of lack of reasonable accommodation in relation to those who have physical disabilities. Without the appropriate learning and teaching approaches, mentorship and guidance, these students face a stressful environment at higher education institutions that could potentially compromise their ability to complete their course work and negatively influence their academic performance. There is scant literature on the experiences of students in South African universities who have invisible disabilities compared with other countries such as Australia, the United Kingdom and the United States of America (Bryant 2014; Mullins & Preyde 2013; Mutanga 2017). Venville et al. (2016) also highlighted that there is a need to address the effectiveness of existing educational support for students with invisible disabilities. Therefore, this study aims to address this gap by exploring the subjective experiences of students with invisible disabilities at a historically disadvantaged university.

## Methods

### Study design and setting

In order to explore the subjective experiences of students with invisible disabilities, this study made use of a qualitative, exploratory research design. This was decided to create a coherent narrative from the perspective of a particular cohort; to understand and represent their experiences and actions as they encounter, engage and live through their situations (Wagner, Kawulich & Garner 2012). Students were recruited from a historically disadvantaged university in the Western Cape.

### Study population and sampling strategy

Participants were recruited using nonprobability sampling methods, specifically, purposive and snowball sampling. A flyer was developed and was initially distributed to the university’s formal disability unit, which provides support for students with disabilities. The invitation was also later opened to the wider university community via the university communication email forum to ensure that all students with invisible disabilities were reached and not only those who were registered with the disability unit. Students were included if they were registered students at the university, had an invisible disability and spoke any three of the Western Cape official languages (English, Afrikaans, IsiXhosa). Students who met the set criteria for this study were emailed information letters and consent forms. Theoretical saturation was reached at 12 interviews. Students were between ages 18 and 50 (mean [*M*] = 33.92; standard deviation [SD] = ± 12.03), where nine participants were not registered and three were registered with the disability unit on campus (OSWD). Most of the students were identified using snowball sampling. Interestingly, many participants did not believe they needed to be registered with a support unit as they did not feel it was necessary.

Table 1 presents the participants’ characteristics. Twelve students (four men and eight women) with invisible disabilities were interviewed. The participants were between the ages of 18 and 50 years. Whilst some of the students were formally registered with the University Disability Unit, others were not. Participants presented with various invisible disabilities as seen here and the length of the diagnosis varied for each participant.

**TABLE 1:** Study participants.

Participant	Age	Gender	Registration status (with university disability unit)	Diagnosis	Length of time since diagnosis (in years)
1	40	Male	Registered	Bipolar disorder, attention deficit hyperactivity disorder (ADHD), depression and anxiety disorder	5
2	50	Male	Not registered	Blind left eye (about 20% peripheral vision), cannot see in 3D	40
3	23	Female	Not registered	Bipolar disorder type 1, generalised anxiety disorder	1
4	41	Female	Registered	Sickle cell disease	Born with it
5	28	Female	Not registered	Autism, bipolar disorder type 2, ADHD	Could not recall
6	50	Female	Not registered	Attention deficit disorder (ADD)	1
7	47	Female	Not registered	Major depressive disorder, anxiety	Could not recall
8	40	Female	Not registered	Bipolar, ADHD, autoimmune	Less than a year
9	18	Male	Not registered	Epilepsy, arthritis	Could not recall
10	20	Female	Not registered	Visual impairment, asthma	Over 5
11	22	Male	Not registered	Anxiety	3
12	28	Female	Registered	Major depressive disorder, borderline personality disorder, generalised anxiety disorder	5

### Data collection and analysis

Semi-structured interviews were conducted online via Google Meet platform from August to September 2020. This was because of the ongoing coronavirus disease 2019 (COVID-19) pandemic, with strict government-mandated lockdown restrictions and safety protocols. Verbal and email consent to record all interviews was sought and obtained from all participants. All interviews were conducted in English as all participants were fluent in this language. Thematic analysis was employed for this study and themes were identified using an inductive approach. The researchers followed the six-step approach as outlined by Clarke and Braun (2013): familiarisation with the data, coding, searching for themes, reviewing themes, defining and naming themes and writing up. All interviews were transcribed by the authors. Although not every author was involved in each interview, all interviews and transcriptions were reviewed by other authors to ensure confirmability.

### Ethical considerations

The researchers received ethical approval to conduct the study from the Humanities and Social Sciences Research Ethics Committee of the University of the Western Cape (HSSREC; HS20/4/49). Approval for conducting research with registered students was also sought from the university’s registrar’s office. Informed consent was obtained, therefore all participants received an information sheet and consent form to review and provide consent to be interviewed. They were also informed that they could decline or stop the interview at any time without prejudice. Should any participant have felt the need for additional support, appropriate referrals would have been provided; however this was not necessary.

## Results

This study set out to explore the subjective experiences of people with invisible disabilities at a historically disadvantaged institution. Findings brought to light significant results concerning the experiences that students with invisible disabilities in higher education are having. There are three thematic categories that appeared within the data, namely:

1. Interpersonal experiences of the student post-diagnosis. The analysis revealed that students are facing various effects of managing an invisible disability both at home and with friends at school.
2. University transition and life experience when students have an invisible disability. This theme explains the experience that students had transitioning into university life, having to disclose their disability and the stigma that may have followed disclosure. Furthermore, the COVID-19 pandemic also had an impact on the student’s LWID experience of higher education.
3. Student experiences of available support for invisible disabilities. Data show that most of the students who were interviewed were not registered with the official university support unit for students with disabilities. There was a lack of awareness regarding the disability unit on campus for some. Furthermore, participants shared their experience with the available support received or not received from peers, lecturers and university staff members.

### The interpersonal experiences of the student post-diagnosis

Amongst the participants, there were a multitude of diagnoses that were considered ‘invisible’, that is, their impediments are not immediately and physically apparent to all. The majority of these diagnoses were psychological in nature, ranging from mood disorders (such as bipolar disorder, major depressive disorder and generalised anxiety disorder) to neuropsychological or behavioural disorders such as autism spectrum disorder and attention deficit (hyperactivity) disorder. In addition, other participants have been diagnosed with epilepsy, arthritis, visual impairments and sickle cell disease.

Each disability is unique and is influenced by a complex interaction of intrapersonal, interpersonal and contextual factors. This ultimately influences the experiences of each person with an invisible disability within the context of their tertiary studies. Some students had been diagnosed as recently as within the 12 months before the interview and others up to 40 years ago. It was evident that the amount of time between their disability and treatment significantly influenced the participants’ level of reflection and engagement. The following themes describe these experiences.

#### Effects of managing an invisible disability

Personal challenges relating to invisible disabilities varied greatly from physical to psychological amongst the participants in the sample. However, there were similarities in some aspects of their experiences in coping with an invisible disability whilst at university, which may not be immediately visible to others. Some reported extreme emotional challenges, with the added physical constraints relating to prescribed medications. For example, participants with bipolar mood disorder and major depressive disorder asserted that their emotional challenges have, at times, been debilitating, owing to the side effects of medications and hospitalisation. This is seen in the given extracts:

‘I think under her care I have attempted suicide about two or three times. But the last time was when she decided to have me committed.’ (Participant 1; 40 [age]; male, mood disorder, bipolar and attention deficit hyperactivity disorder [ADHD], registered)

‘I was hospitalised. I was in a metal health facility for two weeks.’ (Participant 2; 50 [age]; male, visual impairment, not registered)

These diagnoses have resulted in a necessity for them to take medication. Students often had to adhere to prescribed medications that impaired their cognitive and physical abilities. For instance, taking medication to improve their condition often resulted in negative side effects such as lapses in memory, anxiety and inability to concentrate or complete tasks, which caused further emotional distress. This is expressed in the following extracts:

‘You are always on medication. So that’s it. You always get sick, so because of that I was absent from school. It’s been a sick life that’s what I’ll call it.’ (Participant 3; 23 [age]; female, bipolar type 1, generalised anxiety, not registered)

‘… The symptoms were a lot more, so you know thinking like this, there’s like gaps, you forget words, you know, you’d be doing one thing and the next thing you find yourself doing something else….’ (Participant 6; 50 [age]; female; ADD; not registered)

These psychological and physical factors were strongly associated with complex social interactions experienced by participants in their daily lives, such as exchanges with peers, their social lives and experiences at school. This was further exacerbated by the medical costs relating to managing their disability:

‘…I mean, at that time you are growing. I mean, you are 18, 19, 20 and I would see people going out and having a drink and I would want that release but I couldn’t. I couldn’t make myself do normal things….’ (Participant 11; 22 [age]; male; anxiety; not registered)

‘…I was absent from school; I was losing out, you know … I was losing out as a young girl who is always sick at home, that was also a problem on the social cost … and then another challenge is the medical costs, you know, medication is expensive, the medical services are expensive….’ (Participant 4; 41 [age]; female; sickle cell disease; registered)

Students who are unable to engage in daily schooling and social life often have negative social experiences. Some of the experiences related to ridicule experienced from friends and peers for their diagnoses. In addition, not being able to engage in social activities seems to bear on students’ efficacy in completing tasks as well. This is explained as follows:

‘…They would demoralise you; they would mock you because you can’t walk or because you get sick at school. Your friends will not understand. Maybe the teacher only, but your friends they don’t understand….’ (Participant 4; 41 [age]; female; sickle cell disease; registered)

‘[… *I*]f it was something that had an impact on your eye then I couldn’t do it, that was the difficulty, I must admit….’ (Participant 5; 28 [age]; female; autism, bipolar disorder 2; ADHD; not registered)

Although some of these experiences occurred outside of the university environment, it also provides a bit of context in which people with invisible disabilities may reflect and react when entering university life. It provides insight into the life of those who have disabilities which are not immediately perceptible to others. These experiences frames further experiences later on.

### The university transition and life experience when students have an invisible disability

The following section will present the different aspects that influence the student’s experience of higher education. These aspects include the transition process into university and the challenges, both experienced with micro aggressions and disclosure.

#### The transition process

The transition process varied across participants and was influenced by factors such as their self-esteem, being enrolled part-time or full-time, having recently completed high school or having worked before commencing their studies. Students also stated that the different contexts they came from greatly influenced their transition to university.

Contextual factors beyond the university (such as family and community support) which increase or decrease the ability to cope with the demands of tertiary education differed across the participants and impacted the various experiences of students’ transition to university life. The participants in this study expressed that they had difficulties to manage the university workload due to their disabilities, thus leading to procrastination and feelings of anxiety:

‘…I’d never be studying and I’d always panic and leave things to the last minute and be very paranoid and lose it and be completely anxiety-driven and that’s how we operate, you procrastinate until you actually have enough dopamine released to actually do something….’ (Participant 6; 50 [age]; female; ADD; not registered)

‘Academically it was a little bit tough, because with my ADHD I only got diagnosed I think in like my early 30s or something … theory-wise I struggled, the textbooks became overwhelming.’ (Participant 8; 40 [age]; female; bipolar, ADHD, autoimmune; not registered)

#### Perceptions on disclosing their disability

Disclosing an invisible disability was challenging for some participants. for some. These challenges contributed to students’ reluctance to disclose their conditions to lecturers, university staff members and peers. The issue of disclosure is described as ongoing in nature, because they may find themselves having to disclose their disability to various groups of people within the university environment.

Some students opt to not disclose their condition to their lecturers at university if they do not deem it necessary. Whilst some students choose to not disclose their invisible disability, other students experience feelings of frustration and find it difficult to disclose the nature of their condition:

‘[… *I*]t’s very difficult for me to disclose it now … and I don’t know if my behaviour is challenging to the academic and administrative staff and sometimes, you know, I get very frustrated and I’m thinking, is it me or is it the system? And I get quite defensive … and I think, well, is this just about me. And then I don’t want to use it as an excuse but it’s a reality and I don’t know if that reality is dismissible….’ (Participant 6; 50 [age]; female; ADD; not registered)

‘It is really difficult to explain a condition that people don’t see…and something that just starts. It’s been a challenge to explain.’ (Participant 4; 41 [age]; female; sickle cell disease; registered)

These emotions are linked to concerns regarding the way other individuals would receive their diagnosis of their invisible disability and the attitudes and behaviours of those within the university setting towards the student. These concerns and fears are expressed in the following experiences of students:

‘…It’s not really supporting me, I mean, if they are not aware of it, how can they? So they can’t make an overt attempt to support me in any way because there’s been no disclosure….’ (Participant 6; 50 [age]; female; ADD; not registered)

‘…but I haven’t necessarily disclosed it to any of my lecturers in simple terms of like I have bipolar disorder you might not see me sometimes but especially A, he knows sometimes I’m just like I’m not in and then he’ll help out where he can…’ (Participant 3; 23 [age]; female; bipolar type 1, generalised anxiety; not registered)

#### Stigma

Related to disclosure is the challenge of stigma. Students reported the fear of judgement associated with disclosing their conditions. Some students even reported that having an invisible disability was often considered to be taboo and they cautioned against making their conditions known for fear of being perceived and treated differently by others:

‘[…*P*]eople in my support group who advise each other, don’t tell people you have bipolar, and it’s just because of the judgement that comes with it….’ (Participant 8; 40 [age]; female; bipolar, ADHD, autoimmune; not registered)

It is evident that students are deterred from sharing the nature of their disability, which consequently affects the support they may receive within the university setting:

‘…I think it’s a fear of being judged by the next person…like how is this person gonna perceive me….’ (Participant 7; 47 [age]; female; Major Depressive Disorder [MDD], anxiety; not registered)

‘[…*T*]he fact that I don’t even know that my university has support if there is any for people in my circumstances, just says that there is still a stigmatisation about this….’ (Participant 8; 40 [age]; female; bipolar, ADHD, autoimmune; not registered)

Personal and social factors are associated with issues regarding disclosure, which exacerbates the experience of having an invisible disability. This might also relate to the first theme regarding the understanding of having an invisible disability in a personal context.

#### Experiences of learning during a pandemic with an invisible disability

The perceptions held by university staff and students’ peers and the general lack of knowledge regarding invisible disabilities greatly impact students’ willingness to be vocal about their invisible disabilities.

Findings also revealed that the current COVID-19 pandemic had an effect on students’ academic experience. During the lockdown period, it was easy to adjust to the online education platform for some students, but a stressful experience for others who were dependent on in-person contact classes. As such, some students had more positive experiences:

‘[…*I*]f [the university] actually maintained its online presence it would be a big game-changer. It would take a lot of the stress away and the anxiety away from the university experience and day to day….’ (Participant 3; 23 [age]; female; bipolar type 1, generalised anxiety; not registered)

‘…What I have been impressed with is that there’s been a lot of communication during COVID-19 about support and that’s more welcomed. You know that it’s there, and my interest is also around wellness that … it just appears that there aren’t enough opportunities on a wellness level and that seems to have come to fruition during COVID-19, so there’s been a lot more communication from that department….’ (Participant 6; 50 [age]; female; ADD; not registered)

‘[…*T*]he truth … from the university side it’s been a general care for us, apart from the erratic internets, we have been served with some food, soaps … so even though it was not directly coming from the office with students with disabilities, we have been cared for, that talks to COVID-19….’ (Participant 4; 41 [age]; female; sickle cell disease; registered)

On the one hand, some participants perceived the lockdown as a protective factor which buffered the anxieties they experienced being on campus. On the other hand, some participants experienced the national lockdown to have profound negative effects on their mental well-being:

‘…So, I guess also lockdown, being isolated in one place was a huge challenge because I’m used to moving around, I’m used to exercising, I’m a project manager so running around all the time. Then having to be isolated from people and … engagement was … detrimental to how my brain functions….’ (Participant 6; 50 [age]; female; ADD; not registered)

‘[*T*]hat is one of the disadvantages that in facing now I have no human support in my studies anymore … I mean, you just think about how disconnected you feel from an academic society when even every lecture is prerecorded.’ (Participant 8; 40 [age]; female; bipolar, autoimmune, ADHD; not registered)

Although students’ needs were as varied as their invisible disabilities, all students longed for understanding and acceptance from their colleagues and lecturers.

### Student experiences of available support for their invisible disabilities

The participants had various opinions regarding the support they received from the university staff members, lecturers and other peers. Opinions range from students feeling their unique needs were accommodated by the university to others feeling that this was not the case, as some challenges were experienced such as accommodation in the classroom specific to different students’ needs. Within this thematic domain are themes associated with participants’ awareness of the unit, staff members’ awareness and sensitivities towards people with invisible disabilities and the support students’ received or experienced.

#### Lack of awareness of the disability unit

Interestingly, findings suggest that most of the participants were not aware of the disability unit on campus and were not registered. Therefore, these students could not receive the benefits of the disability unit. Similar responses also reflected that some students had heard about the unit, but there was no further interest in formally registering, as seen in the following responses:

‘…No… I am not aware… I just read in one of [*the university’s*] communications about this… just a random email that I saw… but I am not sure where they are on campus, which services they offer or whatever ….’ (Participant 7; 47 [age]; female; Major Depressive Disorder, anxiety; not registered)

‘[…*T*]he fact that I don’t even know that my university has support if there is any for people in my circumstances, just says that there is still a stigmatisation about this….’ (Participant 8; 40 [age]; female; bipolar, ADHD, autoimmune; not registered)

‘[…*O*]kay, they are in the same building as the health services [*OSwD Unit*], right? … I discovered them, not out of my personal interest for that, but I’m also involved or was, I was enquiring about access to the beach for disabled students. Yeah, so I know where it is, like I said, I’ve never considered my condition as a disability … Yeah, so I don’t know what services they offer but I know the department exists….”’ (Participant 6; 50 [age]; female; ADD, not registered)

Other students indicated that they were aware of the disability unit but did not feel the need to use the facilities offered by the unit. Some students also lost hope as they perceived the support offered by the unit as ineffective to their distress:

‘…It was more of a safety net for me, I don’t use any extra time or any of the stuff they offer or really go to the psychologists on campus, I don’t use any of their facilities….’ (Participant 2; 50 [age]; male; blind in left eye, 20% peripheral vision, cannot see in 3D; not registered)

‘…A tutor of mine recommended me to a psychologist. I spoke to someone from ResLife, but there was never a tangible change that I felt. So I lost hope in these people….’ (Participant 11; 22 [age]; male; anxiety; not registered)

It would seem that there were mixed experiences regarding participants’ perceptions, challenges and whether the students required additional support.

#### Academic support

Those participants who were able to access the support unit felt adequately supported and accommodated. Here they expressed satisfaction with the level of accommodation they received from the university; however, it is worth noting that these students are registered with the support unit and have disclosed their invisible disability with staff members and lecturers. As such, for those who have not accessed the official support structures of the university, the stigma attached to disclosure can possibly lead to students not receiving the support they need and ultimately experiencing further challenges within the higher education environment.

Some participants believed that there was a lack of awareness or sensitivity to people with both visible and invisible disabilities. This belief also left participants experiencing a lack of appropriate support from academic and support staff with whom they interacted daily. This may lead to feelings of neglect in students and a negative experience of higher learning or feeling that they do not require as much accommodation as someone whose challenges are more visible:

‘[…*I*]n fact the only place that I don’t get support from for this is in my studies, which is currently actually a big thing.’ (Participant 8; 40 [age]; female; bipolar, ADHD, autoimmune; not registered)

‘…Hence I said I come from a place where everybody understands me. So if the university could offer me a listening ear, that could be accommodative and understanding of the type of person I am so that I can break this chain that has been withholding me from engaging with other people. So I think the university could offer me a unique psychologist that would put everything aside and deal with me and understand me. Because this problem of mine is really hindering me….’ (Participant 11; 22 [age]; male; anxiety; not registered)

‘…Where the university fails is having the necessary instrumentation available for people like myself who are partially sighted and the university also makes the mistake in my case, to assume that I can see….’ (Participant 5; 28 [age]; female; autism, bipolar disorder 2, ADHD; not registered)

Participant 11 was not registered with the disability unit on campus but he was aware of the unit. However, the lack of support does not only exist in the context of higher education but within the individual’s family lives as well. The lack of support experienced by students with an invisible disability seems to be linked to disclosure, where on the one hand if a student does not disclose their invisible disability status, they face feelings associated with lack of support, namely feelings of neglect or being excluded; on the other hand, if students disclose their invisible disability status they face the possibility of stigma. This is a major challenge in the lives of students with an invisible disability in the context of higher education.

## Discussion

Students with invisible disabilities’ transition into university can be challenging. Although certain policies in South Africa advocate for the rights of individuals with disabilities in mainstream education, students often still experience various challenges when they enter the university environment (Matshedisho 2007). Moreover, as Venville et al. (2016) suggested, most existing support structures are developed for those with physical and sensory disabilities. This calls into question a gap in knowledge and approaches to what reasonable accommodation can look like for students with an invisible disability.

As suggested by our findings, there are varying degrees of disabilities, which all require individualised attention, and this is evident in the extracts that have been provided by students. The university’s support unit for students with disabilities on campus serves to accommodate students with disabilities. However, there are some students that are not aware of or choose to not register with this unit on campus, and for this reason have not received any of the benefits that they offer to students with disabilities. For those who were aware of the unit, they did not feel they needed assistance and therefore did not register, or merely used registration as a ‘safety net’ in case they missed classes or tests because of their illness. This is also aligned with the findings of Stone (2005), who qualitatively explored young women’s experiences of living with their invisible disabilities poststroke. The study presents findings that indicate that for some with invisible disabilities, there is a sense that their disability is not ‘as bad as’ those with visible disabilities and only those with visible disabilities are ‘worth taking seriously’ (p. 303).

It is evident that the challenges they experienced might at times be debilitating, owing to the effects of the prescribed medications. Some participants discussed having been prescribed medications to manage symptoms of the disability (Participant 3 and Participant 6) but experienced negative consequences in their academic life, such as lapses in memory, anxiety and inability to concentrate or complete tasks. This also affected the quality of their social interaction with peers and general well-being in consideration of the financial implications and costs of this medication.

When an individual receives a diagnosis for a disability regarded as ‘invisible’, it can significantly impact their academic journey and may unfortunately go unnoticed because it is not ‘obvious’. Should these participants also choose not to register with the university’s support unit, they might face a situation where they have to disclose their disability each time. Participants (specifically participants 7 and 8) expressed that disclosure is an ongoing personal debate as a student. As a result, fear of stigma essentially leads to lack of disclosure and this may negatively impact the academic support, peer support and necessary accommodations and provisions made available for students with invisible disabilities according to their individual needs.

This may be because of a lack of knowledge regarding the purpose of the disability unit and notions of what help may look like in the context of higher education, which facilitated reluctance to use the services. Unfortunately, there are some consequences to not disclosing to the university, such as not receiving the help they need when facing complex challenges on campus and beyond. As mentioned by Participants 6–8 and according to Côté (2009), it is often the fear of stigma and being judged by others that causes an individual to conceal their invisible disability. Furthermore, the general lack of awareness and understanding about invisible disabilities often sets up the possibility of the individual being ‘labelled’ once a disability is disclosed (Shelton & Matthews 2001). Not disclosing one’s invisible disability because of fear of being labelled denies that student from receiving the appropriate guidance and benefits from the disability unit on campus. Therefore, the phenomenon of disclosure is directly linked to the awareness that students have of the disability unit and the potential benefits they can gain from this unit within their academic journey.

Thus, it is imperative that both student and staff (academic and support) are sensitised towards issues of disability in order to avoid possible stigmas or discriminatory behaviours. A common perception of participants was that staff members and lecturers of the university were not aware of certain invisible disabilities and did not know how to appropriately accommodate these students. A lack of awareness amongst staff will directly influence the academic experience of these students as they are not receiving the appropriate support they need to have a fair experience and reach their full potential in the respective courses. There also appeared a lack of awareness amongst fellow students and administration staff. However, the overarching theme was the implications of staff not being equipped with the appropriate knowledge of how to accommodate students with invisible disabilities in their classrooms. Researchers found within the data that participants had opposing views on the amount of support that they receive from the university staff members, lecturers and other peers.

Furthermore, the unforeseen pandemic and national lockdown has forced institutions of learning to embrace blended approaches to learning and teaching. These approaches have been met with enthusiasm by some who have experienced being more supported, given their disability. This type of blended approach (infusing technology and being able to access lecture recordings and notes online) can be beneficial for those who especially suffer from invisible disabilities such as anxiety and attention deficit disorder (ADD) or those who experience learning delays. On the other hand, some participants had a more negative experience as the pandemic brought an increased sense of anxiety regarding their well-being and health. The interviews took place during the first 4 months of the pandemic, which resulted in many features of learning and higher education still being undetermined. Given the South African context, access to stable Internet, data, devices and material remains a significant concern by both students and staff.

Despite the added challenges of the COVID-19 pandemic, some students with invisible disabilities also experienced a lack of support. This appeared to be linked to their willingness, comfort with disclosure and the number of years they have had to process and manage their disability. A student that does not disclose their invisible disability may experience feelings associated with a lack of support, such as neglect. On the other hand, if students disclose their invisible disability status, they face the possibility of stigma from peers and staff members. The stigma attached to disclosure leads to an unhealthy cycle of students not receiving the support they need and ultimately experiencing further challenges within the higher education environment.

## Conclusion

The purpose of this study was to explore the subjective experiences of students with invisible disabilities at a historically disadvantaged university. The experiences and interpersonal challenges put forward by students speak to the challenges that still exist in higher education. Although students’ needs and subjective experiences were as varied as their invisible disabilities, all students in this study longed for understanding and acceptance. The overarching themes were the interpersonal challenges of students with invisible disabilities, their experience of university and the support offered to them by the university. Issues such as lack of awareness, sensitivity, disclosure and stigma remain key factors in the challenges and experiences of students with disabilities in higher education. Thus, it is imperative that individuals are educated and more mindful towards issues of disability to avoid possible stigmatising or discriminatory behaviours against those who have invisible disabilities. More so, this awareness should be evident in both student–staff interactions and in student–peer interactions. In this way, further academic support can be made available to students with disabilities in the university setting. As many international and national policies and bills are available, such as the White Paper on the Rights of Persons with Disabilities (2015), what is now necessary is advocacy for what those policy parameters look like within the context of higher education, and even more so within contexts which might be under-resourced because of sociohistorical legacies.

### Recommendations and limitations of the study

The information collected can be used to inform policies within the university and provide guidelines for improvement within higher education institutions to support students with invisible disabilities. Based on the study’s findings, future research can consider conducting university-wide research to ascertain attitudes or perceptions of students with invisible disabilities. Further research can also potentially focus on intervention efforts to help students with invisible disabilities have a more meaningful university experience. Targeted awareness campaigns can also contribute to a better experience for students with invisible disabilities to ensure that other students, academic and administrative staff are made aware of the possible challenges experienced by people with disabilities not immediately visible (e.g., effects of medication, time required to complete projects etc.).

This study is not without limitations. Firstly, because the study was conducted online and there were time constraints in terms of data collection, processes such as member checks were difficult to complete. Furthermore, because of the volatile nature of Internet connection, the flow of some of the interviews was interrupted by abrupt Internet interferences. This could have had an influence on the information that was shared. Another limitation to this study is that half of the study participants were not registered with the disability unit and thus could not speak to the support that they could have received from the specific unit.
